# HIV self-management perceptions and experiences of students at one university in South Africa

**DOI:** 10.4102/curationis.v47i1.2572

**Published:** 2024-07-31

**Authors:** Siseko Tyabazeka, Wendy Phiri, Regis R. Marie Modeste

**Affiliations:** 1 Department of Nursing Science, Faculty of Health and Wellness, Cape Peninsula University of Technology, Cape Town, South Africa; 2 Department of Nursing and Midwifery, Faculty of Medicine and Health Sciences, Stellenbosch University, Cape Town, South Africa

**Keywords:** university students, experiences, HIV, HIV self-management, young adults

## Abstract

**Background:**

The public health concern posed by HIV in South Africa is significant, particularly among young adults aged 15–34 years. Within this age range, university students present a unique demographic, concurrently managing their HIV condition and academic pursuits, thus raising concerns about HIV management within university environments. Research into the experiences of South African university students living with HIV is relatively sparse.

**Objectives:**

The study aimed to explore the HIV self-management perceptions and experiences of South African university students.

**Method:**

The study employed a qualitative research approach grounded in the constructivist paradigm. Data were collected through semi-structured interviews with eight (8) students living with HIV at a university in the Western Cape area in 2021. Data were analysed through thematic analysis. All ethical principles were adhered to, and trustworthiness was ensured.

**Results:**

Findings revealed that students utilised various strategies to manage their HIV condition, inclusive of maintaining a positive mindset, and adopting a healthy diet. They encountered obstacles such as experiencing HIV-related stigma, which frequently resulted in elevated stress levels. The potential role of peer support groups was also underscored, with students expressing a desire to participate in such groups to maintain their mental health.

**Conclusion:**

Living with HIV is a challenging experience for university students, but self-management of the condition makes life easier for them.

**Contribution:**

These findings contribute to the understanding of HIV self-management perceptions and experiences of university students, and can inform the development of a comprehensive supportive structure that addresses their needs.

## Introduction

Human immunodeficiency virus (HIV) remains a significant public health concern globally, specifically in South Africa. South Africa has the highest number of people living with HIV and is home to the largest antiretroviral therapy (ART) programme in the world (Kim et al. 2021; Manasa et al. 2013). However, the public health concern remains, particularly among South African young adults aged 15–34 years (Stats SA 2022). Within this age range, university students present a unique demographic, concurrently managing their HIV condition and academic endeavours.

Human immunodeficiency virus infection has transitioned from being perceived solely as a fatal disease, to being considered a chronic condition that can be effectively managed with ART (Deeks, Lewin & Havlir 2013; Maartens, Celum & Lewin 2015; Scandlyn 2000). This change necessitates a corresponding shift in patient care approaches, especially concerning self-management. Self-management, in the context of HIV, involves individuals managing symptoms, treatment, physical and psychosocial consequences, and lifestyle changes associated with living with a chronic condition (Schulman-Green et al. 2016). For university students, this responsibility can be intricate because of the confluence of factors such as academic stress, stigma, peer influence and the transition to adulthood (Areri, Marshall & Harvey 2020).

Despite the escalating prevalence of HIV among university students in South Africa, research exploring their distinct experiences and perceptions related to HIV self-management remains scarce (Allinder & Fleischman 2019). To address this gap in the literature, this study was designed to investigate the HIV self-management perceptions and experiences of South African university students living with HIV, providing a more nuanced understanding of their challenges and coping mechanisms.

A good understanding of these experiences is essential in formulating interventions designed to improve the quality of life of students living with HIV and enhance their academic success. Furthermore, such understanding can provide insights into fostering resilience among this vulnerable demographic and promoting a culture of empathy and acceptance within university environments.

## Background

The prevalence of HIV poses a significant public health challenge in South Africa, with an estimated populace of 7.6 million living with the virus by the end of 2021 (UNAIDS 2022). Young adults, particularly those within the 15–34 age bracket, bear the brunt of this epidemic, constituting a significant fraction of new HIV infections (Stats SA 2022). This age group also represents a considerable proportion of the higher education student population, thus raising concerns about HIV management within university environments (Govindasamy et al. 2020).

Typically, university students are in a transition phase marked by identity exploration, burgeoning independence and substantial life changes (Arnett 2016). For those living with chronic conditions such as HIV, these developmental and contextual factors may compound the complexities of HIV self-management (Fegran et al. 2014). In the context of HIV, self-management is acknowledged as a pivotal aspect of living with the condition, encompassing responsibilities such as adherence to medication, symptom and side-effect management, psychological adjustment and navigation of healthcare services (Millard, Elliott & Girdler 2013).

However, research into the experiences of South African university students living with HIV is relatively sparse, with limited comprehension of their unique challenges, needs and coping strategies (Allinder & Fleischman 2019). This knowledge gap inhibits the development of tailored interventions and support services addressing the specific needs of this demographic.

Human immunodeficiency virus management encapsulates a complex process that not only calls for medical intervention but also necessitates psychological, social and behavioural adjustments (Millard et al. 2013). The task of HIV self-management proves particularly challenging for young individuals, given the complexities of navigating adolescence and emerging adulthood, such as identity exploration, emotional development and the cultivation of independence (Arnett 2016). For university students, these developmental challenges intersect with academic pressures, social dynamics and the pursuit of autonomy, creating a unique set of circumstances that could potentially impact their ability to manage their condition effectively (Fegran et al. 2014).

Moreover, persistent issues related to HIV-associated stigma and discrimination exist, even within the realms of higher education settings (Haffejee et al. 2018). These social challenges could potentially compound the difficulties encountered by students living with HIV, affecting their mental health, social relationships and academic performance (Rao et al. 2008). Therefore, comprehending how these students navigate the intricacies of HIV self-management within the university context is crucial.

Within the South African setting, HIV interventions have predominantly focused on prevention measures, testing and access to treatment (UNAIDS 2020). Despite the undeniable importance of these endeavours, a growing recognition exists regarding the necessity to address broader psychosocial and contextual factors that influence individuals’ ability to manage their HIV condition. In the realm of higher education, this manifests as a need to comprehend the specific experiences of university students living with HIV and to develop supportive structures that facilitate their HIV self-management efforts and overall wellbeing (Allinder & Fleischman 2019).

The present study aims to explore the HIV self-management perceptions and experiences of university students living with HIV in South Africa. The findings from the study are anticipated to contribute to a more nuanced understanding of HIV self-management within the university context, ultimately informing strategies and interventions that bolster the health and academic success of students living with HIV.

## Research methods and design

The research employed a qualitative research approach grounded in the constructivist paradigm. This paradigm emphasises the understanding of the world as constructed through the subjective experiences of individuals. Such a paradigm was deemed suitable for the research, given its aim of comprehending the experiences of students living with HIV within a South African university context (Kivunja & Kuyini 2017).

The research was undertaken at a university located in the Western Cape province of South Africa, with a focus on students living with HIV. Data saturation was achieved after conducting interviews with eight (8) participants during the 2021 academic year. Professional referral sampling was applied in this study. Although professional referral sampling has received less notice as a methodology, it is commonly used in health research (Hogan et al. 2009). Professional referral sampling involves selecting respondents through an intermediary who provides professional service to the participants (Hogan et al. 2009), and in this case, it was the professional nurse (PN).

### Study population and sampling strategy

As the topic under investigation was a very sensitive one, the researcher sought permission to conduct interviews and obtained it from the relevant authorities at the campus clinic. The authorities at the campus clinic then referred the researcher to the PN at the HIV unit of the clinic, who works directly with students who are living with HIV. After making initial contact with the PN via email, the researcher then arranged face-to-face meetings with the PN to discuss the protocol and explain the research information sheet to be shared with potential participants. The PN helped identify potential participants and arranged telephonic meetings with them after she explained the study and asked for their consent to participate. Those who agreed to participate also permitted their contact details to be shared with the researcher, who would contact them to arrange interviews. A total of 16 potential participants were contacted in two rounds of recruitment, and data saturation was achieved after eight participants took part in the study. Of the 16 potential participants, two refused to take part in the study mainly because they did not feel comfortable discussing their HIV status or they did not have time because of their academic commitments. The whole recruitment process took about 8 weeks to complete, mainly because of time constraints and study commitments from potential participants.

### Data collection

Data collection was conducted using semi-structured interviews, thus enabling an in-depth exploration of the students’ experiences and perceptions. To be included in the study, participants had to be students living with HIV and attend the campus clinic at the institution, and there were no exclusion criteria as all those who met the inclusion criteria were included in the study if they provided consent. There were two face-to-face interviews and six telephonic interviews, all conducted in English and lasting approximately 30–40 min. The interview sessions were guided by an interview schedule, which offered a conversational framework while allowing flexibility for the participants to articulate their experiences in their own words and for the researcher to probe further as needed. This method facilitated the gathering of rich, detailed data about the students’ experiences (Paradis et al. 2016). The interview guide that the researcher used contained some open-ended questions guided by the study’s objectives and research questions. The research aimed to collect sufficient relevant data to fulfil the study’s requirements. One main question was asked: ‘What are your experiences in your self-management of HIV condition as a higher education student?’ Some follow-up questions and probes included: ‘What are the symptoms that bother you the most?’; ‘How do you manage those symptoms?’; ‘How do you balance your studies with the HIV self-management?’.

### Data analysis

Recorded interviews were transcribed verbatim. Subsequently, data were analysed using thematic analysis, a frequently employed qualitative method that identifies, analyses and reports patterns (themes) within the data (Nowell et al. 2017). This process encompassed six steps: familiarisation with the data, initial code generation, theme searching, theme reviewing, theme defining and naming, and producing the final report (Braun & Clarke 2019). The researcher engaged the services of an independent coder to help with data analysis. The researcher and independent coder conducted their thematic analyses separately and later compared the themes upon which they agreed. They then decided which subthemes to combine into main themes and which to discard. This collaborative approach to thematic analysis was preferable mainly because of its flexibility, which allowed the researcher to gain insight into different perceptions and views.

### Ethical considerations

Ethical considerations were adhered to throughout the study, including obtaining informed consent from the participants, ensuring confidentiality and anonymity, and respecting the participants’ right to withdraw from the study at any point. The researcher also applied for and was granted ethical clearance and institutional permission by Cape Peninsula University of Technology (reference no.: CPUT/HC-REC2021/H12). Participants were given comprehensive information so that participants understood the purpose of the study prior to commencement. Willing participants indicated consent either via the phone or during face-to-face interviews. Participants were also advised that this study was voluntary and that they could decline at any stage. The researcher also took steps within reason to ensure that no harm befell the study participants. Before any of the interviews were conducted, the researcher made it clear to the participants that the interviews would be stopped at any time if the participant felt uncomfortable. Fortunately, no one was either harmed or indicated that they were uncomfortable during the data-gathering process. The researcher also made sure that all the coronavirus disease 2019 (COVID-19) protocols and safety measures were observed at all times in accordance with stipulated precautions in order to safeguard the lives of the participants.

### Trustworthiness

Trustworthiness is an important component in research, and it indicates logical accuracy, being scientifically adequate or trustworthiness of the outcomes concerning strict adherence to the philosophical viewpoint and being meticulous in collecting data (Korstjens & Moser 2018). The researcher took enough care to observe all the required standards of trustworthiness: credibility, dependability, transferability and confirmability. Credibility was ensured through analyst triangulation. Member checking was also done, which involved paraphrasing data from participants to ascertain that the meaning drawn by the researcher was actually what the participant had just said. Transferability was ensured through thick descriptions and comparisons of results from empirical data with findings from related literature. Furthermore, as noted by Stahl and King (2020), in this study, the triangulation, peer debriefing and ensuring audit trail also ensured confirmability and dependability.

## Results

As illustrated in Table 1, the majority of the participants were female (seven out of the eight participants), and included those in the undergraduate and postgraduate programmes. The participants were from different faculties within the university and their ages ranged from 21 to 32, representing the general university population. Other studies on university students in South Africa show roughly a similar age range to the university population (Bor, Musakwa & Onoya 2021; Gumindega & Maharaj 2022; Haffejee et al. 2018).

**TABLE 1:** Demographic details of participants.

Participant number	Age (years)	Gender	Level of study	Programme of study
1	27	Female	Fourth-year (undergraduate)	Electrical engineering
2	21	Female	Second year (undergraduate)	Nursing
3	23	Female	Third year (undergraduate)	Nursing
4	32	Female	Fourth-year (undergraduate)	Civil engineering
5	24	Female	First year (postgraduate)	Architecture
6	25	Female	First year (postgraduate)	Electrical engineering
7	27	Female	First year (postgraduate)	Mechanical engineering
8	24	Male	Fourth-year (undergraduate)	Land and construction architecture

The research findings presented below are arranged into key thematic areas that emerged during the data analysis process. They are also supported by available literature which was used to make comparisons with evidence already out. The themes included improved understanding of the virus, living with HIV, recognition of HIV stigma, the HIV self-management journey and positive self-management strategies. The themes are illustrated in Table 2.

**TABLE 2:** Themes and subthemes.

Objectives	Main themes	Subthemes
To explore higher education students’ understanding of their HIV condition	1. Improved understanding of the virus	1.1 Demystifying the virus. 1.2. Accepting one’s HIV status
	2. Living with HIV	2.1 HIV effects on social life 2.2 Disruption of normal academic routine
	3. Recognition of HIV stigma	3.1 Perceived and experienced stigma 3.2 Broken trust and apportioning blame
To explore the experiences of higher education students on their HIV self-management at a higher education institution	4. The HIV self-management journey	4.1 Challenges in self-management of HIV 4.2 Support in HIV self-management
	5. Positive self-management strategies	5.1 Maintenance of good physical health 5.2 Having a positive mindset

### Improved understanding of the virus

The first theme in the study highlights an improved understanding of HIV, with subthemes covering the demystifying of the virus and acceptance of HIV status. Prior to the HIV diagnosis, the participants reported that their understanding of the virus was not based on real facts to a very large extent. The participants relied more on myths and hearsay even though several awareness campaigns were conducted on campus. These myths created the impression that having HIV was akin to a death sentence, hence there was a lot of fear and stigma around HIV issues. The situation changed once the participants were diagnosed with HIV because they began to access new information about the virus from counsellors, nurses and from their research, which removed the myths they previously had. One participant said the following:

‘You know the problem with people, they think when you are HIV positive, and you have AIDS, which is not. HIV is manageable.’ (Participant 5, female, 24 years)

This new information brought clarity and helped to break the myths that shaped their previous perceptions of HIV as further explained and illustrated by another participant:

‘… I am HIV positive, and my partner is HIV negative. It does not mean he will be positive. I can have children that are HIV-negative because there are pills that you take when you are pregnant to protect your child.’ (Participant 4, female, 32 years)

The demystification was important, as the participants became more knowledgeable about HIV; it became easier for them to accept their status and adopt steps towards managing the virus, as remarked by one of the participants:

‘HIV has now become my lifestyle; I have accepted the condition, and I do get stressed out like any other student but being HIV positive does not affect my studies at all … Well … since I have been attending counselling since 2015, I think it’s getting easier with time.’ (Participant 6, female, 25 years)

### Living with HIV

Most of the participants in the study indicated that living with HIV as a university student brought with it several challenges. Two subthemes that emerged under this main theme were HIV effects on social life and disruption of normal academic routine. Participants indicated that their social lives were disrupted in several ways, such as isolation from close friends and classmates as a consequence of living with the virus. One participant said the following about how her social interaction with her fellow classmates and friends was affected by her HIV condition:

‘So there was this other day at our high school residence that one of my roommates opened my bag and saw the medication that I was taking, and she showed it to a lot of other learners all over the school. People started looking at me differently … I felt like I was left out alone; I would cry for no reason.’ (Participant 2, female, 21 years)

Living with HIV has also made it difficult for some participants to maintain or create new intimate relationships, and some relationships broke down after their partners discovered they were HIV positive. For example, Participant 3 explained that it has become very difficult for her to have a boyfriend since she contracted the virus, and this was understood as a result of fear and a lack of understanding on the part of the potential boyfriends:

‘Ever since I was diagnosed with HIV, it has not been easy to have a boyfriend because you open up to that person about your condition; they just stigmatise you and don’t want to be with you. I don’t blame them; surely, they are scared of their health (scared of getting HIV infection).’ (Participant 3, female, 23 years)

Participants also noted that living with HIV has also disrupted their normal study routines with some negative outcomes at times. Participant 6 explained that her whole academic routine was disturbed to the extent that she performed dismally because her condition had overwhelmed her:

‘That year I failed 50% of my modules because I was not coping emotionally and psychologically and that was not a good year for me, so I had to redo the modules in 2016.’ (Participant 6, female, 25 years)

It is quite unfortunate that some measures that are meant to manage the virus and promote a healthy livelihood for the participants had an adverse impact on their studies. Participants pointed out that the timetables they followed in taking their HIV medication often affected their study times. Participant 1 said the following with regard to her medications and how they affected her study times:

‘It’s quite difficult when you are a student, and you don’t have a certain pattern that you have, so at times you find out that you have to study at 2 am and you take the pills maybe at 9 pm, and the side effects start kicking in. That was my main challenge in taking my medication.’ (Participant 1, female, 27 years)

Similarly, another participant regularly missed her early classes because of the side effects of her medication:

‘With that, I will oversleep and end up missing the morning classes and only getting the after-tea classes that start at 11 am.’ (Participant 4, female, 32 years)

### Recognition of HIV stigma

The HIV stigma is still very rife and the participants in this study indicated that they also practised it before they were diagnosed. The subthemes that emerged under this theme were perceived and experienced stigma, broken trust and apportioning blame. Human immunodeficiency virus stigma occurred in several ways, such as saying unpleasant things about those living with the virus, posting derogatory messages on social media platforms and direct verbal abuse aimed at those living with HIV. One participant indicated that stigma was rife on campus and a lot of hurtful things were said about those diagnosed with the virus:

‘Yes, there is stigma at university when you get sick, the other student would say “he or she’s been shocked by electricity” meaning that the person is HIV positive looking at their lifestyle. I think they don’t have enough knowledge about HIV.’ (Participant 7, female, 27 years)

As the participants became infected with the virus, they have become more conscious of the stigma around HIV among their peers. While they experience it personally, some of it is perceived. They quickly recognise it when it occurs and from the things that are said by some of their peers, and it is quite traumatic for them in most cases, as indicated by some participants:

‘Yes, there is quite a lot of stigma around the HIV condition.’ (Participant 6, female, 25 years)

‘There is a lot of stigma that is going on around the concept of HIV.’ (Participant 8, male, 24 years)

In this modern world where social media platforms have become places of daily interactions, participants also indicated that HIV stigma had also become a common occurrence on these platforms, which was quite devastating for those living with HIV. Some people took advantage of the anonymity they enjoyed on these platforms to post horrible and abusive content towards people living with HIV:

‘I got here, and the clinic was about to move from the off-campus clinic to the campus clinic, and there were all these horrible, horrible comments on Facebook about how … they refer to people living with the condition as if we are invalid, as if you do not matter, you’re not human.’ (Participant 1, female, 27 years)

For most participants, sustaining an intimate relationship with the person responsible for infecting them was difficult. The lack of trust and realisation of stigma was also noted beyond the intimate partners, as participants indicated the inability to trust their friends who displayed HIV-related stigma. Participant 6 highlighted the fact that after she was diagnosed, her relations with her friends were affected because she could not trust them enough to disclose her condition to them:

‘I had to change friends at that time because I did not trust them enough to tell them about my condition as I feared that they would judge me.’ (Participant 6, female, 25 years)

As if the stigma they faced was not enough, most participants grappled with understanding their HIV status soon after finding out they were positive and they also had questions about how they came to contract the virus. In cases where participants were in relationships, they blamed their partners, who isolated them, and the trust that kept them together was broken. For example, after Participant 3 was diagnosed, she blamed her boyfriend because she believed that he was the one who had infected her, and in that case, she felt betrayed and broke up with him:

‘Yes, I have. In 2018, when I got diagnosed, I was having a boyfriend, the one that infected me so I couldn’t be with him anymore, so we broke up.’ (Participant 3, female, 23 years)

In cases where the HIV infection was perinatally acquired, a similar blame was directed to parents, and this was also noted in this study as illustrated by the following quote:

‘I felt like I was left out alone; I would cry for no reason. It made me blame my parents; it made me view life differently at that point. The question I was asking was; why me? What I hated most was that my mother knew that she was HIV positive when she gave birth to me, and she knew there were pills to take to make sure that I don’t get positive; I have a big brother that passed away due to HIV right after birth. My mother knew that my brother died from HIV, but when she fell pregnant with me, she did not protect me … so that created a lot of anger in me.’ (Participant 2, female, 21 years)

### The HIV self-management journey

As reported by the participants in this study, living with HIV is similar to embarking on a journey that is filled with both positive and negative experiences. The two subthemes that emerged under this theme were challenges in the self-management of HIV and support in HIV self-management. From the information shared by the participants, their HIV experiences required emotional strength, a positive mindset and support from loved ones and their communities. This was mainly because their condition exposed them to various challenges they had to deal with on a daily basis. As illustrated by the next quote, some participants lived with the fear of disclosing their status to friends and fellow students because of the stigma and discrimination it brought:

‘The way our residence is built, it has dividing cupboards in such a way that the other person would not see what is happening on the other side. I then always made sure that my medication was on the side where she would not see it, and when I took it, I made sure that I did not make any noise as I did not want her to hear anything and start questioning me on why I take pills every day.’ (Participant 3, female, 23 years)

The stress associated with keeping their HIV status a secret, taking their medication in private and dealing with the side effects of their medication was also a source of anxiety for some participants:

‘Every time when I am not here on campus, I have to worry about having to hide my medication, even when I go out with friends on weekends away.’ (Participant 3, female, 23 years)

The side effects of the medication itself were also cited as a challenge by some participants, which sometimes affected consistency in taking them. This posed a greater risk to their health, but as indicated by Participant 6, it was a way to minimise the terrible experiences of the side effects:

‘Also, because of the side effects that I had, mostly nausea, I would skip a day purposely just to bypass the side effects. I know exactly that would compromise my health, but the side effects were terrible.’ (Participant 6, female, 25 years)

Despite the numerous challenges cited by the participants in relation to their HIV status, some of them also enjoyed varying levels of support from close relations, friends, fellow students and their partners. One participant indicated that her family was very supportive:

‘I was fortunate enough to get support from my family.’ (Participant 4, female, 32 years)

Similarly, even grandmothers were quite supportive in some cases:

‘My grandmother would go fetch the medication for me and send it to me.’ (Participant 2, female, 21 years)

In most cases, the participants who indicated that they enjoyed social support were those who had managed to disclose their status to their close friends and loved ones.

### Positive self-management strategies

The identification of methods utilised by higher education students to manage their HIV status revealed that students living with HIV employed several strategies for condition management. The subthemes that emerged from the data were maintaining good physical health and having a positive attitude.

From the interviews conducted, participants highlighted that their HIV self-management experiences involved engaging in activities that were aimed at ensuring that they maintained good physical health. That included participating in activities enhancing their physical fitness and avoiding anything perceived as interfering with their wellbeing. This was illustrated by Participant 8, who stated:

‘A change in living and eating habits was necessitated, alongside regular exercise.’ (Participant 8, male, 24 years)

Another participant stated that:

‘I joined the gym as well, so I am maintaining my weight, and it’s not out of control.’ (Participant 7, female, 27 years)

Participants also highlighted their heightened awareness of their wellbeing since they got diagnosed. In addition, a deeper understanding of their HIV status prompted them to make changes, such as dietary adjustments that would benefit their health. For instance, Participant 3 stated:

‘With dizziness, I cut on fatty food and made sure in the mornings I don’t just jump off the bed when I wake up, I first rest and then wake up gently.’ (Participant 3, female, 23 years)

Besides sticking to specific diets and regular physical exercise, some participants changed their lifestyle and stopped certain unhealthy habits they used to engage in before they were diagnosed:

‘I used to party a lot, I had to change that, and now I don’t have the energy to party anymore, and I stopped drinking alcohol.’ (Participant 4, female, 32 years)

Most participants realised that the more they understood how the virus worked, the more they could focus on adopting measures that improved their wellbeing. It was out of this understanding that they realised the importance of keeping a positive mindset which was crucial to their overall wellbeing and enabled them in their HIV self-management practices and ongoing experiences. This was illustrated by a participant who stated:

‘After I was diagnosed, I learned that there is more to life than just sitting at home and being sick. With HIV, you can go back to society and engage and be like any other person, as long as you take your medication.’ (Participant 8, male, 24 years)

Similarly, Participant 6 was able to self-introspect, and she realised that her life and health were more important than the virus she carried. This positive mindset allowed her to value herself and develop a great sense of self-belief, leading her to concentrate on her studies:

‘When I found out about my status, I told myself that I am more important than anything and that I will not let anyone bring any negativity to me because my health is more important. I was not stressed about it…I realised that I am not a failure, so it was self-motivation. I needed to believe in myself. I even told myself that after this qualification, I want to do my masters in Civil engineering.’ (Participant 4, female, 32 years)

Participants indicated different ways of achieving this positive mindset include finding hobbies, keeping themselves busy with work and engaging in fun activities with close friends instead of stressing about their HIV-positive status:

‘I just don’t want to think about it because it frustrates me. I just have to keep myself busy.’ (Participant 3, female, 23 years)

The researcher noted that some of the activities mentioned were not new to them, but what changed was that they now understood the purpose for which they were engaging, which was to maintain positive health outcomes and avoid things that could compromise their wellbeing as their status had changed.

## Discussion

These findings furnish vital insights into the perceptions and experiences of university students living with HIV in one university in South Africa, shedding light on the complexities of HIV self-management within this context. Prior to the HIV diagnosis, the participants reported that their understanding of the virus was not based on real facts to a very large extent. Instead, the participants relied more on myths and hearsay, even though several awareness campaigns were conducted on campus. These myths created the impression that having HIV was akin to a death sentence; hence, there was a lot of fear and stigma around HIV issues. This limited knowledge of HIV among youth has also been documented in a South African study by De Wet, Akinyemi and Odimegwu (2019), who noted that only about 10% of youth affected by HIV had 100% accurate knowledge. In this study, the situation changed once the participants were diagnosed with HIV because they began accessing new information about the virus from counsellors, nurses and their own personal research. This new information brought clarity and helped to break the myths that shaped their previous perceptions of HIV.

Most of the participants in the study indicated that living with HIV as a university student brought several challenges in different areas of their lives. Participants indicated that the most prominent effect of living with HIV was its disruption to their academic and social lives. This experienced disruption has been documented in previous studies. For example, a study by Payán et al. (2019) in the Dominican Republic on young women diagnosed with HIV revealed that the virus disrupted several aspects of their lives, such as their social and work lives. Similarly, another study by Wouters and De Wet (2016), which investigated the experiences of women living with HIV in South Africa, also indicated that some of the participants’ lives were disrupted by the side effects they experienced from taking ART medicines. While ARTs are generally well-tolerated, potential side effects such as fatigue, nausea or mood changes can impact daily life as reported in this study. In this study, managing these side effects alongside academic responsibilities was also reported as challenging, and linked to disturbances in academic life and commitment, leading the students to miss some of the academic activities. This challenging situation is not unique among the participants in the study. Ankrah et al. (2016) also confirmed some of these challenges faced by students living with HIV such as difficulties in taking medicines on time, failure to keep up with study times and side effects from medicines. When facing such challenges, the participants in this study developed plans to achieve better health outcomes. This highlights that the participants developed resilience similar to what has been documented by Dulin et al. (2018), and such resilience facilitated their HIV journey. Resilience at different levels such as individual, interpersonal and community levels has been noted to be beneficial for people living with HIV (PLWHIV) (Dulin et al. 2018).

However, for most participants, developing plans and strategies to make the necessary changes in response to these disruptions took a while. Participants indicated that they had to make significant life changes, including stopping certain activities such as drinking alcohol and in some cases, they lost friends and partners because of living with the virus, similar to what has been reported by Ankrah et al. (2016). Making lifestyle changes after an HIV diagnosis is beneficial if changes are geared towards a healthy lifestyle, and literature has documented that such changes include engaging in physical exercises and making dietary changes (Arias-Colmenero et al. 2020). A study of factors affecting the quality of life of PLWHIV in Uganda confirmed that there are more benefits in adopting positive changes (Mutabazi-Mwesigire et al. 2015).

In this study, some participants indicated that they had disclosed their HIV status to family and close friends, and those who could not, had to grapple with maintaining their friendships while concealing any evidence about their status from these friends. A review of both qualitative and quantitative studies conducted by Evangeli and Wroe (2017) revealed that there are perceived interpersonal risks associated with HIV disclosure that are mainly motivated by fear, anxiety and worry. Despite the numerous challenges cited by the participants in relation to their HIV status, they also enjoyed varying levels of support from close relations, friends, fellow students and their partners. According to Hogwood, Campbell and Butler (2012), the benefits of HIV status disclosure outweigh the negatives, including receiving effective treatment and having a higher chance of being supported by loved ones and partners. On the other hand, it has been noted that despite the benefits of HIV disclosure, it can also become a cause for social isolation when those disclosed to do not accept the person living with HIV (Arias-Colmenero et al. 2020). Furthermore, Arrey et al. (2015) confirm this challenge in disclosure, as they indicated that disclosure remains a major challenge for HIV patients in general. For the participants in this study, the non-disclosure meant having to conceal medications and other treatments. Living with HIV has also made it difficult for some participants to maintain or create new intimate relationships, as the partners or even potential partners may stigmatise them. Rejection was also reported, and some relationships broke down after their partners discovered they were HIV positive. This fits the findings from a Canadian study with women living with HIV highlighting that HIV stigma continues to be a barrier to engaging in safe and healthy relationships for those living with HIV (Carter et al. 2019). Similarly, Psaros et al. (2012) noted that most women living with HIV experienced challenges in engaging in intimate relationships, and fear of disclosure and stigma were some of the main sources of their non-engagement in intimate relationships.

Another important finding from this study was that, at this institution, HIV stigma is still very rife among university students and the participants in this study indicated that they also practised it before they were diagnosed. Stigma occurred in several ways, such as saying unpleasant things about those living with the virus, posting derogatory messages on social media platforms and direct verbal abuse aimed at those living with HIV. This indicates the different manifestations of HIV stigma, fitting with the reports by Azher (2018) that stigma manifests itself in several ways and emanates from different groups, including in the family, in relationships, in the community, in education, at work, in religion and even in healthcare workers. Unsurprisingly, participants indicated that, in most cases, they lived in constant fear of being stigmatised by their fellow students because of their HIV status. These results are similar to the results of a study conducted by Azher (2018) on PLWHIV in India, which noted that once people are diagnosed with HIV, they immediately become afraid of discrimination and stigma from relatives, friends and their communities. Such fears are not unfounded, as another study in South Africa noted multiple types of discrimination towards adolescents living with HIV (Pantelic et al. 2020). In support of the findings from this study, it has also been shown that in some cases, these fears were mainly based on what they had witnessed happening to other people living with the virus (Matthew et al. 2019). Discrimination towards people living with HIV interferes with their HIV self-management as it reduces their access to healthcare services (Pantelic et al. 2020). In South African communities, Mokgatle and Madiba (2023) have noted that despite the various interventions around HIV infection and its treatment including the fight against stigma, stigma remains a problem, and might be explained by the limited holistic understanding of how HIV is transmitted and constant fear of HIV infection. In this study, the participants reported the common stigma combined with limited knowledge about HIV, which even applied to them prior to their diagnosis. The university still needs to put more effort into HIV awareness for students, which can reduce the perceived and experienced stigma that was noted by the participants in this study.

On the other hand, some participants still struggled with the circumstances surrounding how they contracted the virus. Even though they were more knowledgeable about the virus, some participants had lost trust in their partners, whom they blamed for infecting them with the virus. This resulted in broken relationships and an extra emotional burden that was not good for their wellbeing. Similar findings have been reported in a study conducted by Fauk et al. (2022) on women living with HIV in low and middle-income Asian countries. The findings of the study by Fauk et al. (2022) showed that it was common among women to experience fear, self-blame, blaming spouses and feelings of anger for contracting HIV. Self-blame was normally associated with those who felt that they did not heed their parents’ advice against engaging in unprotected sex (Fauk et al. 2022).

This study also established that living with HIV is like a journey that one embarks on, which exposes one to different experiences, both positive and negative. The participants’ HIV experiences required emotional strength, a positive mindset and support from loved ones and their communities. Having the virus meant that they had to be more aware of their health than before they were diagnosed. This heightened awareness of oneself after an HIV diagnosis has been noted in previous studies. Some participants reported that they had to adhere to their treatment schedules and deal with the side effects of medicines which sometimes meant missing some of their classes or study times or even missing submission deadlines for assignments and projects.

### Recommendations

After analysing the primary data that were collected, this study has the following recommendations for different stakeholders based on the findings that were made.

#### Students can form HIV awareness clubs that are open to all for the purposes of spreading awareness and tackling HIV stigma

As students are able to form different social clubs on campus, they can form HIV awareness clubs which can be platforms for organising different activities such as plays, quizzes and competitions that are aimed at tackling stigma and spreading positive information to dispel myths about HIV.

#### More research is required to investigate the levels of HIV stigma on university campuses and explore ways to reduce it

The participants in this study agreed that stigma was still rife, and it was quite challenging for them to disclose their HIV status to close friends and classmates. This was quite stressful because they were forced to do normal things such as taking their medicines in hiding.

#### Researchers should also investigate the effectiveness of current HIV self-management strategies

This study investigated the HIV self-management experiences of students living with HIV, and some HIV self-management strategies that the students implement have been presented, although their effectiveness remains to be established. Therefore, their effectiveness needs to be measured under different environments and situations to establish key standards that can be recommended for other persons living with HIV.

#### The university should provide support (such as funding) for more research in strategies that can enhance current models of HIV education

This study has established that HIV stigma is still common among HIV-free students, even though there are programmes to teach students about HIV and raise awareness of how it can be managed. It is important to measure the effectiveness of these programmes and establish if more needs to be done to improve them.

#### More support, such as flexible timelines for the submission of academic assignments, is required for students living with HIV to minimise disruptions to their academic programmes

Participants noted that they experience disruptions to their academic programmes because of the schedules they are supposed to follow in taking their medicines and the side effects they experience. Therefore, allowing them more time and flexibility to submit their assignments might help them cope with their challenges, which could enhance their HIV self-management.

#### More research is required to investigate the self-management initiatives of students living with HIV at more universities

This study was a qualitative one, and it focused on a single institution, which means the findings are only limited to this one institution and cannot be generalised. A study with a wider scope to cover more universities and which uses a mixed methods approach would provide more details about the self-management initiatives of more students living with HIV while its findings can be generalised. This will also help to add more literature on this subject which is currently very limited.

### Limitations

The major limitation of this research was that it was conducted when South Africa was affected by the COVID-19 pandemic, and lockdown measures were still in place, with limited movement for everyone. This also impacted the selection of participants, which was already difficult because of the sensitivity of the subject under discussion. The limited movement may have created some bias in the selection of participants, as only those who were able to come to campus could be selected for the study, leaving out the other group of students who might have provided a different narrative. The other limitation was the fact that only those who were accessing the campus clinic could be included in the study, because of the sampling technique that was employed, and those not coming to the clinic and attending community clinics or private health practitioners could have enriched the findings of the study.

## Conclusion

The study investigated the perceptions and experiences of university students living with HIV in South Africa, concentrating on understanding their self-management strategies and challenges. Employing a qualitative research approach grounded in the constructivist paradigm, semi-structured interviews with students living with HIV at a South African university were conducted. Findings revealed that students utilised various strategies to manage their HIV condition, inclusive of maintaining a positive mindset, engaging in physical exercise and adopting a healthy diet. They encountered obstacles such as dealing with the side effects of ARTs and experiencing HIV-related stigma, which frequently resulted in elevated stress levels. These findings contribute to the understanding of HIV self-management in the university context, informing the development of comprehensive support structures that address the multifaceted needs of university students living with HIV.
